# Male Age Influences Re-mating Incidence and Sperm Use in Females of the Dengue Vector *Aedes aegypti*

**DOI:** 10.3389/fphys.2021.691221

**Published:** 2021-07-01

**Authors:** Juliana Agudelo, Catalina Alfonso-Parra, Frank W. Avila

**Affiliations:** ^1^Max Planck Tandem Group in Mosquito Reproductive Biology, Universidad de Antioquia, Medellín, Colombia; ^2^Instituto Colombiano de Medicina Tropical, Universidad CES, Sabaneta, Colombia

**Keywords:** sperm competition, re-mating, sperm transfer, reproduction, male senescence, post-mating

## Abstract

Diseases transmitted by female *Aedes aegypti* mosquitoes are public health issues in countries in the tropics and sub-tropics. As in other insects, *A. aegypti* females undergo behavioral and physiological changes upon mating that principally act to facilitate the production of progeny. The primary effectors of *A. aegypti* female post-mating responses are male-derived seminal proteins that are transferred to females during mating. Increased male age reduces ejaculate function in numerous taxa and alters seminal protein composition in *Drosophila melanogaster*, but the impacts of male age on female *A. aegypti* post-mating responses are unknown. Here, we used “old” (21–22 days old) and “young” (4–5 days old) *A. aegypti* males to assess the influence of male age on oviposition, fertility, and re-mating incidence in their mates. We also examined how age influenced paternity share in females initially mated to young or old males that subsequently re-mated with a transgenic male that transferred RFP-labeled sperm and whose progeny inherited a larval-expressed GFP marker. We found that increased male age had no effect on female fecundity or fertility but significantly impacted their ability to prevent re-mating in their mates—more than half (54.5%) of the females mated to an old male re-mated, compared to 24% of females initially mated to a young male. Polyandrous *A. aegypti* females displayed first male precedence regardless of the age of their initial mate. However, young males were better able to compete with rival male sperm, siring significantly more progeny (77%) compared to old males (64%). Young males had significantly more sperm in their seminal vesicles than old males at the time of mating, although males of both age groups transferred similar numbers of sperm to their mates. Our results suggest that male senescence differentially impacts the induction of some post-mating changes in *A. aegypti* females. As the effect of age may be further exacerbated in the field, age-related declines in male ability to induce sexual refractoriness have implications for *A. aegypti* population control programs that release adults into the environment.

## Introduction

In insects, mating initiates a series of physiological and behavioral changes in females. Seminal fluid proteins (SFPs), transferred along with sperm to the female reproductive tract during mating, are the major effectors of female post-mating changes in several insect species. SFPs can stimulate egg development, ovulation, oviposition, and alter female life span (Avila et al., 2011; Hopkins et al., 2018). Further, SFPs are often required for sperm accumulation into the storage organs and the subsequent release of sperm to fertilize eggs (Avila et al., 2010; Avila and Wolfner, 2017). A key behavioral change mediated by SFPs is the severe reduction in sexual receptivity to subsequent male suitors (Chapman et al., 2003; Helinski et al., 2012a).

Female *Aedes aegypti* mosquitoes—vectors of the dengue (Guzman and Harris, 2015), Zika (Alfonso-Parra and Avila, 2018) and yellow fever viruses (Chippaux and Chippaux, 2018), among others—display significantly reduced sexual receptivity shortly after mating (Degner and Harrington, 2016a). The reduction in sexual receptivity is mediated by SFPs produced in the accessory gland of the male reproductive tract (Craig, 1967; Helinski et al., 2012a). However, given the opportunity, ~25% of *A. aegypti* females re-mate within 2 h of an initial mating (Degner and Harrington, 2016a). Re-mating incidence decreases with an increasing post-mating interval; *A. aegypti* females are completely refractory to an additional mating by 24 h and re-insemination is not observed after this time (Degner and Harrington, 2016a). The SFP head peptide-I contributes to the short-term suppression of re-mating in *A. aegypti* (Duvall et al., 2017), but the SFP that inhibits re-mating in the long-term has not been identified to date. In addition to SFP-induced re-mating inhibition, *A. aegypti* females require SFPs in aggregate to lay a normal complement of eggs after consuming a blood meal (Villarreal et al., 2018) even though vitellogenesis in anautogenous mosquitoes begins after blood ingestion (Attardo et al., 2005).

Factors that alter ejaculate function may impact female post-mating responses, including the inhibition of additional mating, the induction of egg laying and/or female sperm use. A major factor that can influence post-mating responses is male age, as male senescence reduces ejaculate function in several taxa (Johnson et al., 2015; Fricke and Koppik, 2019). For example, mates of old *Drosophila melanogaster* males are less fertile and are more likely to re-mate compared to mates of young males (Koppik and Fricke, 2017; Ruhmann et al., 2018), and old males of the *Allonemobius socius* complex are less able than young males to induce egg laying in their mates (Marshall et al., 2009). However, old males sometimes appear superior to their younger counterparts in eliciting female post-mating responses. Old *Ostrinia nubilalis* males are better able to inhibit re-mating in their mates compared to young males (Milonas et al., 2017), while in *Drosophila bipectinata* (Santhosh and Krishna, 2013) and *Dermestes maculatus* (Jones and Elgar, 2004), mates of old males lay more eggs than mates of young males. Although male age is likely to influence induction of *A. aegypti* female post-mating responses, this area has not been fully explored to date.

The effect of male age on female post-mating responses may be related to changes in SFP function and/or the efficiency of SFP transfer to females during mating. SFP composition in the *D. melanogaster* male accessory glands, the primary site of SFP synthesis in this species (Avila et al., 2011; Hopkins et al., 2018), is altered as males age—the abundance of some SFPs increases while others remain unchanged (Sepil et al., 2020). Further, old *D. melanogaster* males are defective at transferring SFPs despite a higher SFP abundance compared to young males (Sepil et al., 2020). Protein concentration also increases with age in the accessory glands of old *D. bipectinata* (Santhosh and Krishna, 2013) and *Anastrepha ludens* males (Herrera-Cruz et al., 2018). However, in *A. socius*, an SFP that induces egg laying decreases in abundance as males age (Marshall et al., 2009).

Paternity share is also influenced by male age in female insects that re-mate (Jones et al., 2007; Ruhmann et al., 2018). Competition for fertilization opportunities between rival males within the female reproductive tract is a consequence of female re-mating, as sperm may be stored from each sexual encounter. The presence of sperm from more than one male often results in differential fertilization of female eggs. Sperm use patterns in multiply mated females have been extensively studied in *D. melanogaster*, where the last mating male sires the majority of progeny (Lefevre and Jonsson, 1962; Clark et al., 1995). However, last male precedence is influenced by genetic and environmental factors (Singh et al., 2002; Schnakenberg et al., 2012). The number of copulations and the time interval between each mating event (Laturney et al., 2018) also influence sperm competition outcomes. Although SFPs influence sperm competition outcomes (Avila et al., 2011), declines in sperm function are also likely to affect female sperm use. While *A. aegypti* females that mate with more than one male produce mixed progeny (Carvalho et al., 2018), the proportion of sperm used from each sexual encounter, and the effect of male age on paternity share, has not been reported.

In this study, we assessed the influence of male age on female post-mating responses in *A. aegypti*. We examined re-mating incidence, egg-laying, and fertility, and determined sperm use patterns in multiply mated females after an initial mating to an old or young male. As sperm transfer by *A. aegypti* males is reported to increase with age (Ponlawat and Harrington, 2009), old males may increase fertilization opportunities by transferring larger sperm quantities to their mates. Therefore, we also quantified sperm from the seminal vesicles, the organ that stores mature sperm that will be transferred in the ejaculate (Foster and Lea, 1975), before and after mating in males of both age groups to determine the quantity of sperm transferred to their mates. Because male size influences the number of sperm transferred to females (Ponlawat and Harrington, 2007, 2009), and female size influences sperm uptake into the spermathecae (De Jesus and Reiskind, 2016), we controlled for adult size to minimize these effects. Our results highlight how increased age of *A. aegypti* males may alter female post-mating responses in this disease vector and have implications for contemporary control methods that release transgenic (Qsim et al., 2017) or *Wolbachia*-infected adults into the field (O’Neill et al., 2018), as an inability to suppress re-mating by liberated males may influence the successful implementation of these programs.

## Materials and Methods

### Mosquitoes

We used two different *A. aegypti* strains in our assays: Thai and DsRed. Thai strain *A. aegypti* was collected in Bangkok, Thailand and has been maintained in colony since 2009. The DsRed strain contains two transgenes: one that labels sperm with the red fluorescent protein DsRed (Aaβ2t::DsRed) and another that expresses green fluorescent protein (GFP) in the larval eyes of both sexes (3PXP::GFP). This transgenic strain has been maintained in colony since its creation (Smith et al., 2007). Mosquito eggs were hatched under vacuum pressure (−50 kPa) and larvae reared at a density of 200/l in purified water. We supplemented each rearing container with four large (7.2–8.2 mm) Hikari Gold Cichlid food pellets (Hikari, Himeji, Japan). This rearing method consistently generates adults of similar sizes (League et al., 2019; Ramírez-Sanchez et al., 2020). Pupae were transferred to 5 ml tubes to ensure virginity, and resulting adults separated into sex-specific cages upon eclosion. Larvae and adults were maintained in an incubator at 27°C and 80% relative humidity. Adults had constant access to 10% sucrose. Four–five-day-old adults were used in all experiments, with the exception of “old” males, which were aged for 21–22 days. Wing lengths were measured as in Heuvel (1963) to estimate individual size.

### Mating and Re-mating Assays

For our fertility assays, a single virgin Thai strain male and female were placed into a 22 × 22 × 24 cm cage until a copulation occurred, defined as genitalia engagement for ≥10 s (Alfonso-Parra et al., 2014; Ramírez-Sanchez et al., 2020); matings with old and young males were performed in parallel. For our re-mating assays, Thai strain females were first mated to a young or old Thai strain male in parallel, and then given the opportunity to re-mate with a DsRed strain male. We observed the first mating until a copulation occurred. After uncoupling, females were immediately aspirated into a 22 × 22 × 24 cm cage with 25 DsRed males until a 1:1 male–female ratio was reached, the cage was then placed into the incubator for 4 h, after which males were removed. Two independent biological replicates were performed for all assays. For our fertility assays, females were blood-fed on the arm of a volunteer shortly after the observed mating and again 6 days later to obtain eggs from two oviposition cycles. For our re-mating assays, females were blood-fed 24 h later. Blood feeding on human subjects was approved by the Bioethics Committee Sede de Investigación Universitaria (University of Antioquia) and volunteers signed a consent form. Identification of females that re-mated was conducted after oviposition assays (see below) by dissection of the lower reproductive tract in 1X PBS; the presence (re-mated) or absence (not re-mated) of fluorescent sperm was determined using a Nikon Eclipse Ti-U fluorescent microscope.

### Oviposition Assays and Paternity Determination

Four days after blood-feeding, females were individually aspirated into 50 ml conical tubes with a 13 cm × 4 cm paper towel strip and 6.5 ml of purified water and given an identification code. After 48 h, the paper strip was removed, and the eggs were counted using a Zeiss Stemi 508 stereo microscope. Eggs were partially dried and stored in the environmental chamber until hatching 5–7 days later. Eggs were hatched by placing the egg strip into a 40 ml cup filled with purified water, adding a pinch of active yeast, and applying vacuum pressure for 30 min. Resulting larvae were counted 4–6 days after hatching. Hatch percentage was calculated as # of larvae/# of eggs (females that laid zero eggs were excluded from the analysis). To determine paternity in multiply mated females, third instar larvae were placed into a 24-well cell culture plate (two per well) and visualized with a Nikon Eclipse Ti-U fluorescent microscope to assess the presence (sired by the DsRed male) or absence (sired by the Thai male) of GFP expression from the larval eye.

### Sperm Quantification

Virgin and mated males from each age group were knocked down on ice shortly after mating assays and stored at –80°C until dissection. Sperm were quantified from the seminal vesicles using a modified protocol from Ponlawat and Harrington, 2007. Briefly, seminal vesicles and associated accessory glands were dissected in 1X PBS. Dissected tissue was placed into a 200 μl chamber containing 100 μl of 1X PBS, ruptured with minutiae pins to release sperm and mixed by pipetting up and down. An additional 100 μl of PBS was added and the solution re-mixed. Ten 5 μl aliquots of the sperm mixture were placed into a glass slide and dried at 50°C for 5 min. Sperm were fixed in 70% ethanol and stained with Giemsa dye (Merck, Kenilworth, United States). Sperm heads in each drop were counted under brightfield illumination at 200X magnification. This subsample was used to calculate total sperm.

### Statistical Analysis

R statistical software version 3.6.1 coupled with RStudio version 1.2.1335 (RStudio_Team, 2020) was used for all analyses. We first evaluated if each replicate of our assays differed in the characteristics we were examining. For fecundity and seminal vesicle sperm quantity, a linear model was developed, using mating status and replicate as fixed variables. For resulting larvae and hatch percentage, a generalized linear model was used. Because no significant differences were found between replicates, data from these two experiments were combined and replicate used as a random variable in the models. Data from fecundity and resulting larvae were first analyzed to determine the probability distribution that fit the data including normal, negative binomial, and Poisson distributions. The Akaike information criterion (AIC) was used to compare the best distribution that fit the data, where the lowest AIC value corresponds to the best fitted distribution.

To evaluate fecundity, we used male age and female mating status (singly or multiply mated) as fixed variables and replicate as a random variable in a linear mixed model (LMM). To evaluate progeny sired by each male, and the interaction with male age, we used a generalized linear mixed model (GLMM) with a binomial distribution using progeny and male age (old and young) as fixed variables in the model. The proportion of larvae from each male in relation to total larvae was used as the response variable. Differences in P1 between old and young males were evaluated with a *post hoc* Tukey test. Hatch percentage was analyzed as the total number of larvae from both males in relation to the total number of eggs using male age as a fixed variable and replicates as a random variable in a GLMM with a binomial distribution. Sperm quantity between old and young virgin and once mated males was evaluated using a LMM with male age and mating status as fixed variables in the model. Finally, we assessed the sizes of all adults used in our re-mating assays: the size of males used in each assay (old Thai, young Thai, and DsRed), and differences between replicates, was evaluated using a linear model (LM) with male age and replicates as fixed variables. Female size between replicates was evaluated using a LM with replicate as a fixed factor in the model. Re-mating incidence was evaluated by performing the chi-square test of independence based on the contingency table of two variables—male age (old and young) and mating status (re-mated and not re-mated)—using the R stats package and chi-square test function (this was developed for each replicate and both replicates combined).

## Results

### Male Age Does Not Influence Fecundity or Fertility of *A. aegypti* Females

**Figure 1:**
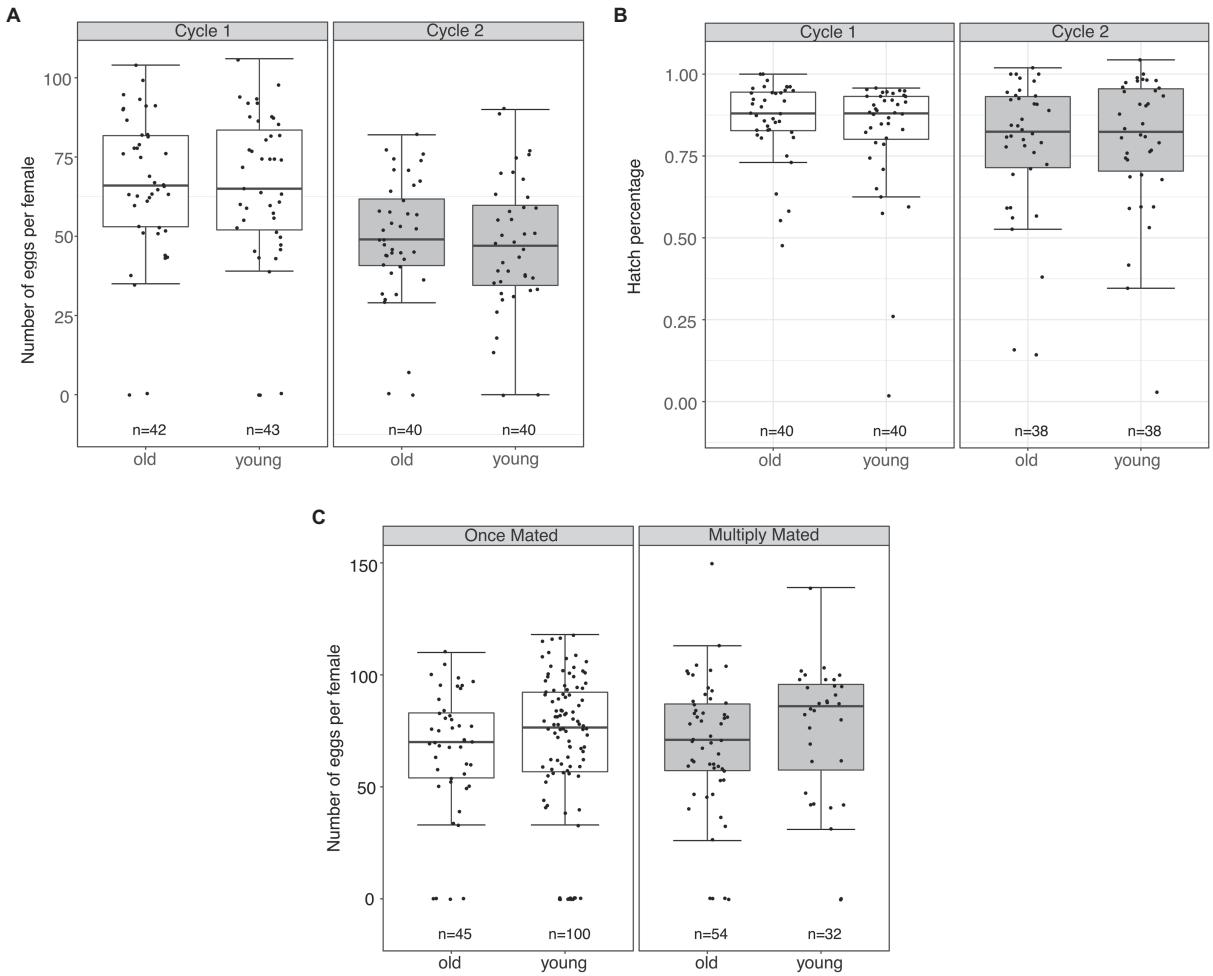
Male age does not influence fecundity or fertility of *Aedes aegypti* females. **(A)** Total eggs laid for the first two oviposition cycles from females mated to old or young males. **(B)** Hatch percentage of eggs from the first two oviposition cycles from females mated to old or young males. **(C)** Total eggs laid by singly mated females to old or young males (left panel), and multiply mated females initially mated to old or young males (right panel). The middle horizontal line in the box plots represents the median, the lower and upper margins of the box represent the 25th and 75th quartile, and the whiskers extend to the minimum and maximum of the data (excluding outliers, shown as points outside the whiskers).

We initially assessed fecundity and fertility of the first two oviposition cycles in females mated to an old or young male. We found that females mated to a young or old male produced a similar number of eggs for two oviposition cycles (LMM: Df = 1, *F* = 0.206, *p* = 0.65; Figure 1A). Further, male age did not influence hatch percentage in the first two cycles of oviposition (GLMM: Df = 1, *F* = 0.778, *p* = 0.379; Figure 1B).

### The Age of *A. aegypti* Males Influences Female Re-mating Incidence

We next examined if the ability of *A. aegypti* males to induce sexual refractoriness in their mates is altered with increased age. We observed a significant correlation between the age of the initial male and the proportion of females that re-mated [*χ*^2^(1, *N* = 231) = 22.23, *p* < 0.00001]. When mated first to a young male, a quarter of the females re-mated (Table 1). However, when mated first to an old male, more than half of the females re-mated (Table 1).

**Table 1:** Female re-mating incidence is influenced by male age in *Aedes aegypti.*

	Age of first mating male	% of females that re-mated	*N*
Replicate 1	Old	56%	41
*χ^2^* (1, *N* = 113) = 10.92, *p* < 0.0009	Young	25%	72
Replicate 2	Old	53%	58
*χ^2^* (1, *N* = 118) = 11.33, *p* < 0.0076	Young	23%	60

We next examined fecundity in females from our re-mating assays, as we were able to identify (1) singly mated females to young or old males and (2) multiply mated females initially mated to a young or old male (see section “Materials and Methods”). In singly mated females, male age did not influence fecundity (LMM: Df = 1, *F* = 0.55, *p =* 0.45; Figure 1C, left panel). Re-mating also had no effect on the overall number of eggs laid by females of either group (LMM: Df = 1, *F* = 0.9, *p =* 0.34; Figure 1C, right panel). Further, hatch percentage of multiply mated females did not differ when initially mated to a male of either age group (GLMM: Df = 1, *F* = 0.67, *p =* 0.50; Supplementary Figure S1).

### *Aedes aegypti* Females Display First Male Precedence

**Figure 2:**
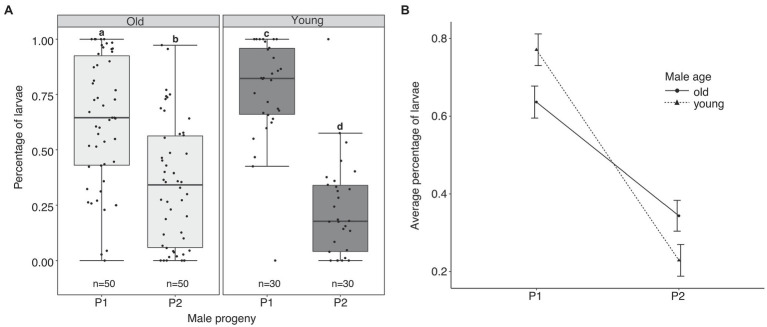
Sperm use patterns in multiply mated *A. aegypti* females. **(A)** The proportion of progeny sired by the first (P1) and second (P2) mating male for each age group. The middle horizontal line in the box plots represents the median, the lower and upper margins of the box represent the 25th and 75th quartiles, and the whiskers extend to the minimum and maximum of the data (excluding outliers, shown as points outside the whiskers). Different letters above each box plot represent significant differences (*p* < 0.05) for a Tukey test. **(B)** Mean (±SE) P1 and P2 scores in females initially mated to an old or young male.

We next examined resulting progeny of multiply mated females after their first oviposition cycle to determine if paternity share is influenced by male age. We found that irrespective of the age of their first mate, *A. aegypti* females displayed first male precedence (Figures 2A,B). However, we observed a significant interaction of male age on the proportion of progeny sired by the first mating male (P1; GLMM: Df = 3, *F* = 46.42, *p* < 0.0001; Figure 2B)—young males sired significantly more progeny than old males in a sperm competitive environment (77 and 64%, respectively).

### Old and Young Males Transfer Similar Quantities of Sperm to Their Mates

**Figure 3:**
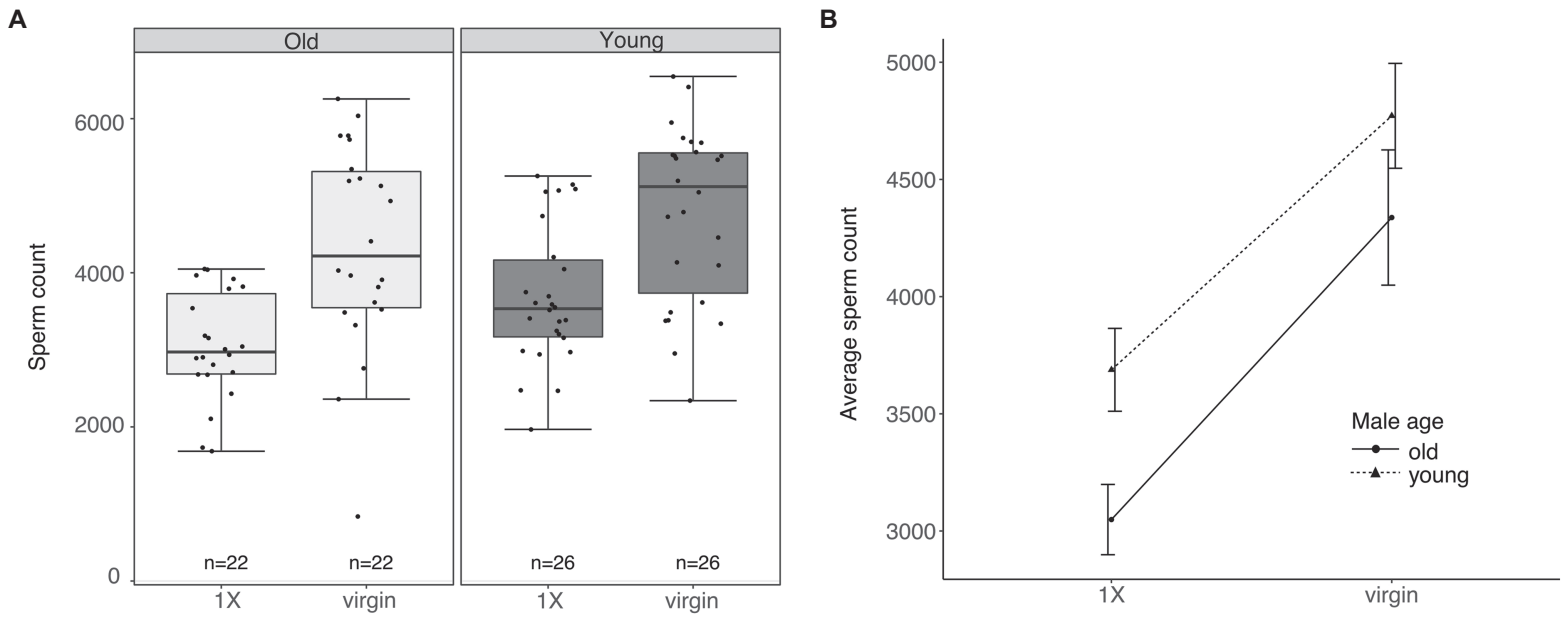
Old and young males transfer similar amounts of sperm to their mates. **(A)** Total sperm contained in the seminal vesicles of virgin and once mated (1X) males of both age groups. The middle horizontal line in the box plots represents the median, the lower and upper margins of the box represent the 25th and 75th quartiles, and the whiskers extend to the minimum and maximum of the data (excluding outliers, shown as points outside the whiskers). **(B)** Mean (±SE) seminal vesicle sperm in virgin and 1X males of both age groups.

We quantified sperm from the seminal vesicles of virgin and mated males to assess the number of sperm transferred by old and young males to their mates during mating. We found that young males contained significantly more sperm in their seminal vesicles compared to old males (LMM: Df = 1, *F* = 6.20, *p =* 0.014; Figures 3A,B). However, we did not observe a significant interaction of age and mating status on seminal vesicle sperm quantities (LMM: Df = 1, *F* = 0.22, *p =* 0.635; Figure 3B).

Finally, we assessed body size of the adults used in our re-mating assays. Using wing length to estimate body size, we found that the size of the males used in our assays did not differ between replicates (LM: Df = 1, *F* = 0.28, *p =* 0.59; Supplementary Figure S2). Although DsRed males were significantly larger than Thai males (Supplementary Figure S2), they were similarly sized in both replicates. Further, Thai males, both young and old, were similarly sized in both replicates, and no significant size differences between the two male age groups were observed (Supplementary Figure S2). Female size also did not differ between replicates (LM: Df = 1, *F* = 1.08, *p =* 0.3027; Supplementary Figure S3).

## Discussion

The induction of female post-mating responses is an essential step in the successful production of progeny. In numerous insect species, the primary effectors of physiological and behavioral changes observed in mated females are male-derived SFPs (Avila et al., 2011; Hopkins et al., 2018). Several SFP-induced changes appear to facilitate the production of progeny, such as structural changes of the female reproductive tract that prepare the female for egg laying (Mattei et al., 2015) or changes in feeding behaviors related to increased egg production (Carvalho et al., 2006). However, others are more selfish from the male perspective, such as the SFP-induced inhibition of female re-mating (Chapman et al., 2003; Helinski et al., 2012a), which reduces the threat of sperm competition. Although *A. aegypti* females are considered monandrous, a proportion re-mate within hours of an initial mating in the laboratory (Degner and Harrington, 2016a), and re-mating has been observed in semi-field conditions (Helinski et al., 2012b) and in a natural population (Richardson et al., 2015). Factors that reduce the quantity of SFPs transferred during mating, such as successive mating by males (Alfonso-Parra et al., 2014), can impact female fertility and the ability of males to prevent re-mating in their mates (Helinski and Harrington, 2011; Ramírez-Sanchez et al., 2020). Here, we assessed how the age of males influenced fecundity, fertility, re-mating incidence, and sperm use in *A. aegypti* females.

Increased age of *A. aegypti* males significantly impacted their ability to prevent re-mating in their mates but did not influence resulting fecundity or fertility, suggesting that SFPs may be differentially affected by male age in *A. aegypti*. Differing effects of male age on female post-mating responses are observed in a number of insects. Aging is likely to impact the function and/or concentration of male-derived seminal components that mediate female post-mating responses. In *D. melanogaster* (Sepil et al., 2020), *D. bipectinata* (Santhosh and Krishna, 2013), *A. ludens* (Herrera-Cruz et al., 2018), and *A. socius* (Marshall et al., 2009), aging alters protein concentration in the male accessory glands, a possible culprit of the changes in female post-mating responses observed upon mating with old males. However, defects in ejaculate transfer may also play a role in the reduction of SFP-induced changes, an effect of age reported in *D. melanogaster* (Sepil et al., 2020). It should be noted that seminal components are not always the primary effectors of female post-mating responses, such as in *A. ludens*, where accessory gland proteins do not inhibit female re-mating (Herrera-Cruz et al., 2018), with female age influencing re-mating incidence in this species (Abraham et al., 2016). Further exploration is required to identify how male age alters SFP composition and/or transfer in *A. aegypti*, and the effect of age on the other female post-mating responses, such as increased host-seeking behavior (Lee and Klowden, 1999), sperm storage (Degner and Harrington, 2016b), and/or transcriptional changes in female reproductive tract tissues that follow mating (Alfonso-Parra et al., 2016; Camargo et al., 2020; Pascini et al., 2020).

Seminal fluid also mediates sperm competition in some insect species (den Boer et al., 2010; Wigby et al., 2020), and SFPs can influence sperm competition outcomes (Avila and Wolfner, 2009; Avila et al., 2010). Young *A. aegypti* males sired more progeny in a sperm competitive environment compared to old males, similar to results observed in *D. melanogaster* (Ruhmann et al., 2018), suggesting that young ejaculates better compete with rival males. This difference may be attributable to an age-related decline in SFP function, sperm function, or both. As the time interval between mating events influences female sperm use (Laturney et al., 2018), young males may be better able to prevent female re-mating in the short-term, leading to better sperm competition outcomes. Moreover, in *D. melanogaster*, sperm quantities in the female storage organs influence sperm use after a subsequent mating (Avila and Wolfner, 2009). As SFPs effect sperm accumulation into the female storage organs (Avila and Wolfner, 2017), old males may be sub-optimal at inducing efficient sperm storage in their mates, which has been observed in *D. melanogaster* (Ruhmann et al., 2018). Functional declines in SFPs that mediate sperm storage may be mitigated by a second mating male, as SFP receipt upon a subsequent insemination can complement ejaculate defects of the first male in polyandrous females (Xue and Noll, 2000; Misra and Wolfner, 2020).

Changes in sperm quantity within the male reproductive tract also accompany aging in males of several insect species. Sperm quantity in the male reproductive tract of *A. albopictus* (Hatala et al., 2018), *Ceratitis capitata* (Costa et al., 2012), and *A. ludens* (Reyes-Hernández and Pérez-Staples, 2017) increases with age. Old *C. capitata* males also transfer larger sperm quantities to their mates (Costa et al., 2012). We found that although old *A. aegypti* males had reduced sperm quantities in their seminal vesicles, they transferred a similar number of sperm to their mates. In field collected *A. aegypti*, 10-day-old males transferred significantly more sperm to females compared to 1-day-old males (Ponlawat and Harrington, 2009). However, the difference between age groups was significantly reduced in laboratory-reared males, suggesting that age-related effects of sperm transfer are minimized when males are reared and maintained under optimal conditions. Sperm appear to maintain their function in the *A. aegypti* male reproductive tract as males age, as females primarily utilized sperm from the first mating male in both age groups and mates of old males were similarly fertile to mates of young males. However, sperm production may be impacted as males age, as is observed in *Drosophila* (Sepil et al., 2020), a potential effect of age not assessed in our study.

## Conclusion

Declines in ejaculate function can seriously alter the induction of female post-mating responses. In the disease vector *A. aegypti*, identifying factors that alter female post-mating responses are important for population control programs that release modified males into the environment. Mating with males with reduced SFP function may permit additional mating opportunities for females, undermining the successful implementation of these programs. We show that increased age of *A. aegypti* males simultaneously impairs their ability to induce female refractoriness and to compete with rival male sperm. The effects of male age we observed in the laboratory may be further exacerbated in the field, where conditions are often sub-optimal. The impact of male age on control programs that exclusively release males into the field can be minimized by liberating males shortly after sexual maturation. However, male age may play a larger role in population replacement programs where liberated adults are expected to become entrenched in the field, with modified males expected to be maintained long term. Our results, along with those of others, suggest that mating with substandard males (e.g., old, small-sized, or SFP-depleted) may be common in the field, which can lead to polyandrous *A. aegypti* females.

## Data Availability Statement

The raw data supporting the conclusions of this article will be made available by the authors, without undue reservation.

## Ethics Statement

Blood feeding on human subjects was approved by the Bioethics Committee Sede de Investigación Universitaria (University of Antioquia) and volunteers signed a consent form.

## Author Contributions

FA and CA-P conceived and designed the experiments. FA analyzed the data and wrote the manuscript. JA, CA-P, and FA performed the experiments and reviewed the manuscript. All authors contributed to the article and approved the submitted version.

### Conflict of Interest

The authors declare that the research was conducted in the absence of any commercial or financial relationships that could be construed as a potential conflict of interest.
