# Supplementary material for: Male Age Influences Re-mating Incidence and Sperm Use in Females of the Dengue Vector Aedes aegypti
Source: Front Physiol. 2021 Jul 1;12:691221. doi: 10.3389/fphys.2021.691221 (PMC8329734; doi:10.3389/fphys.2021.691221)
Supplement: Supplementary file 1 [file Table_1.DOCX]

Supplementary Material


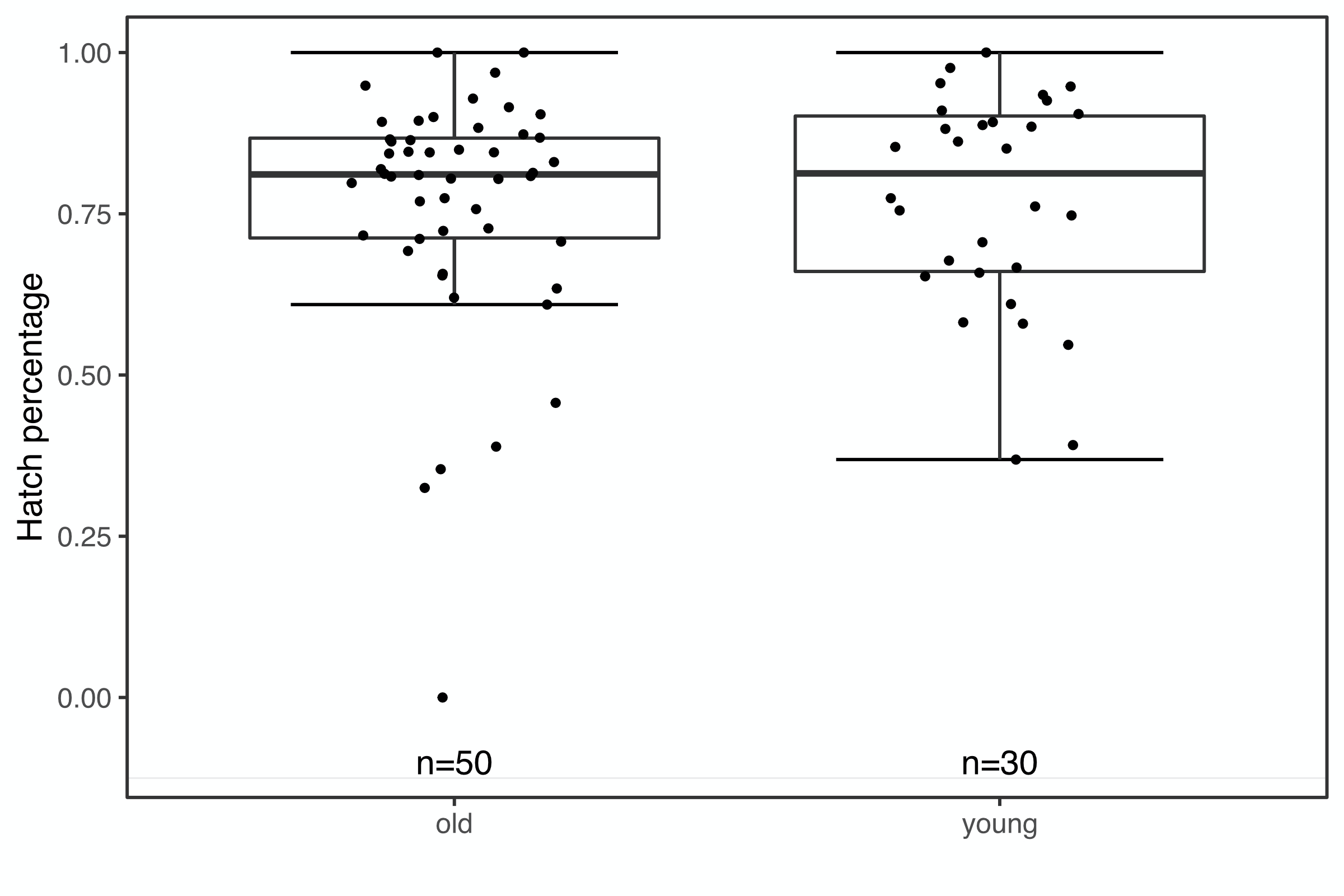


**Figure S1**. Hatch percentage of multiply mated females initially mated to an old or young male.

**
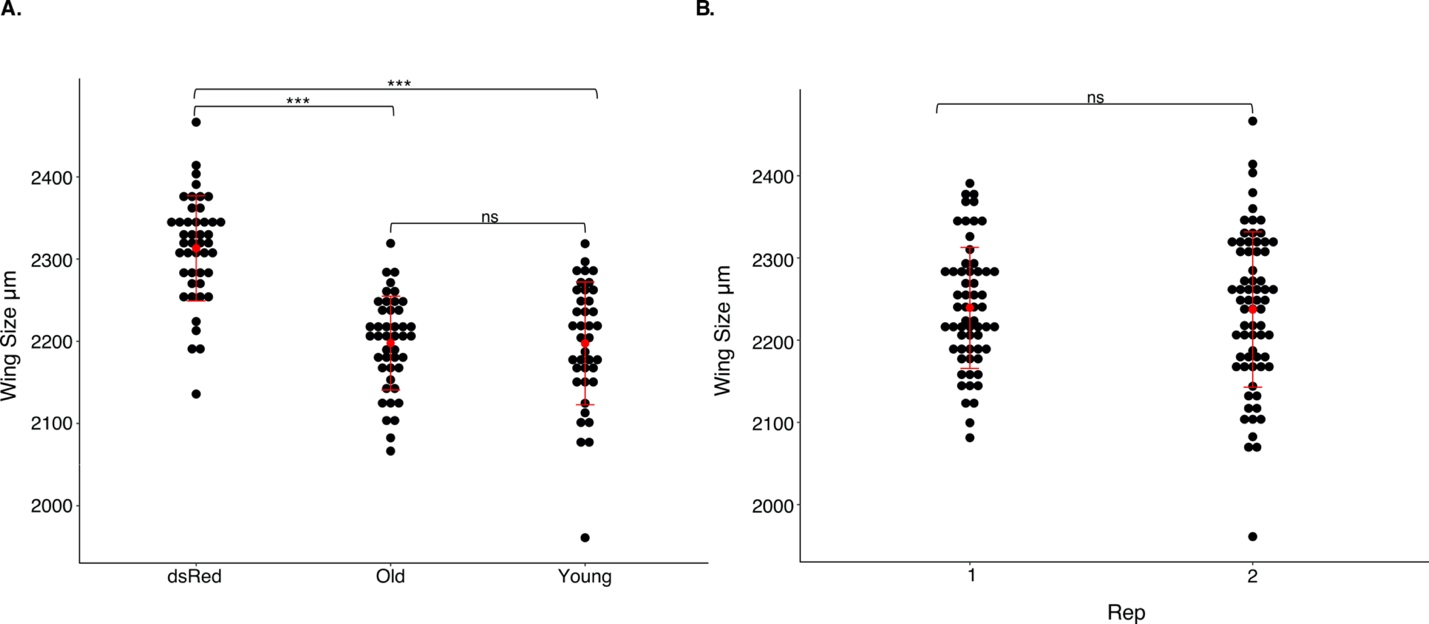
**

**Figure S2.** Sizes of males used in our re-mating assays. Size of old and young Thai males, and DsRed males (**A**). Overall male sizes in replicate 1 and 2 (**B**). Males were similarly sized in each replicate (LM: Df = 2, F = 1.47, *p* = 0.59). ****p* < 0.001 for a Tukey-test; n.s. = not significant.

**
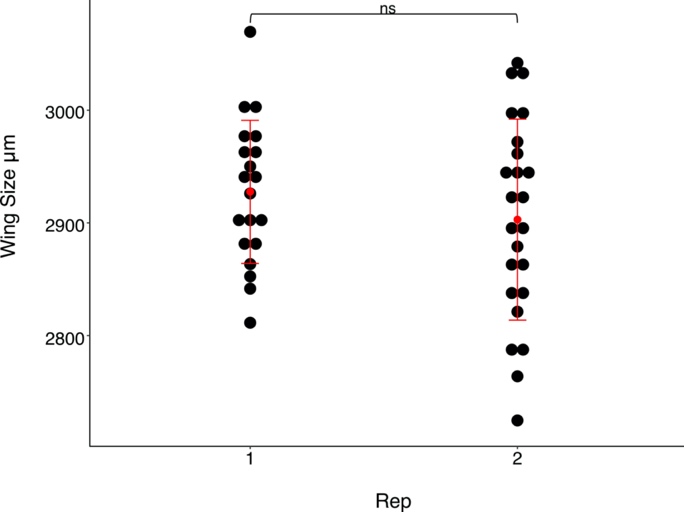
**

**Figure S3.** Size of Thai strain females used in our re-mating assays. No differences were observed in female size between replicates (LM: Df = 1, F = 1.08, *p* = 0.3027); n.s. = not significant.
